# The second study of infectious intestinal disease (IID2): increased rates of recurrent diarrhoea in individuals aged 65 years and above

**DOI:** 10.1186/1471-2458-13-739

**Published:** 2013-08-09

**Authors:** Clarence C Tam, Laura Viviani, Laura C Rodrigues, Sarah J O’Brien

**Affiliations:** 1 Department of Infectious Disease Epidemiology, London School of Hygiene & Tropical Medicine, Keppel Street, London WC1E 7HT, UK; 2 Saw Swee Hock School of Public Health, National University of Singapore, Singapore, Singapore; 3 University of Liverpool Institute of Infection and Global Health, National Consortium for Zoonosis Research, Leahurst Campus, Chester High Road, Neston CH64 7TE, South Wirral, UK

**Keywords:** Diarrhoea, Diarrhoeal diseases, Infectious intestinal disease, Enteric pathogens, Elderly populations, Cohort studies

## Abstract

**Background:**

Infectious intestinal disease (IID) is a major health and economic burden in high-income countries. In the UK, there are an estimated 17 million IID cases annually, of which 6 million are caused by the 12 most common pathogens. Host factors that influence risk of IID are not well understood.

**Methods:**

We analyzed data from the IID2 Study, a UK cohort that measured IID incidence, to investigate factors associated with recurrent IID. We calculated rates of IID by age group, sex, previous episodes experienced, and socioecomic indicators. We used Cox models to investigate factors associated with recurrent illness.

**Results:**

The rate of IID was five times higher among infants than those aged 65 years and above (hazard ratio, HR = 5.0, 95% CI: 3.1 – 8.0). However, the association between previous IID and a subsequent IID episode was stronger in the elderly. Among those aged 65 years and above, each additional IID episode increased the rate of subsequent IID three-fold (HR = 3.1, 95% CI: 2.5 – 3.7). Among infants, the corresponding increase was 1.7-fold (HR = 1.7, 95% CI: 1.3 – 2.3).

**Conclusions:**

Elderly populations have a high propensity for recurrent IID. More detailed studies are needed to identify vulnerable subgroups and susceptibility factors, and inform adequate control policies among the elderly.

## Background

Infectious intestinal disease (IID) is a syndrome characterised by symptoms of diarrhoea and/or vomiting, often accompanied by additional symptoms including fever and abdominal pain. It is caused by a wide range of pathogens or their associated toxins, and infection with these agents commonly results from the ingestion of contaminated foods, through the faeco-oral route, or from zoonotic transmission. Disease is generally mild and of short duration, but can result in severe illness, hospitalisation and even death, while some pathogens are associated with long-term sequelae, including haemolytic uraemic syndrome, Guillain-Barré syndrome and postinfectious arthritis.

IID is a major cause of morbidity in high-income countries. In the UK, the incidence of IID was recently estimated in the Second Study of Infectious Intestinal Disease (IID2), a prospective study to measure the rate and microbial aetiology of IID occurring in the general population, presenting to primary care, and reported to national surveillance. In that study, the incidence of all-cause IID was estimated at 274 cases per 1000 person-years, equating to approximately 17 million cases and 1 million general practice (GP) consultations each year [1]; among thosed aged 65 years and above, the estimated rate was 195 cases per 1000 person-years (95% CI: 173 – 220) [2]. In two large national studies conducted in Australia in 2001 and 2008 to determine incidence and outcomes of gastroenteritis in people aged over 65 years, the estimated rate of illness was 330 episodes of gastroenteritis per 1,000 person years (95% confidence interval (CI): 240 – 420) [3]. In England and Wales, more than 60,000 laboratory-confirmed campylobacteriosis cases are reported each year; persons over 65 account for 20% of reported cases. The corresponding percentages are 11% for salmonellosis, 55% for listeriosis and 72% for norovirus IID, the latter resulting primarily from outbreaks in institutions and healthcare settings [4]. In addition, *Clostridium difficile* is one of the most commonly studied causes of gastroenteritis in the elderly. Morbidity and mortality associated with *Clostridium difficile* infection is an important problem among vulnerable and elderly hospitalised populations [5], although data on community carriage levels, i.e. in people residing at home and not in long term care facilities, are scarce. In the IID2 Study, this pathogen did not feature among the causative agents of IID in a general population cohort, and was responsible for 0.1% of cases presenting to general practice [6]. In one study in Merseyside, England, 4% of healthy elderly patients living at home were found to be excreting *C. difficile*. All were symptom free [7].

In recent years, there have been major changes in the epidemiology of infectious intestinal and foodborne diseases in the elderly, including campylobacteriosis [8] and listeriosis [9]. Indeed, the incidence of several major pathogens reported to national surveillance systems has increased in people over the age of 60 years. What is not always clear from analyses of routine national surveillance data, which are often anonymised, is whether or not the increased incidence represents more people affected overall or an increase in people with repeat infections. The former might indicate that exposure to foodborne pathogens has changed whilst the latter might indicate an increase in susceptibility amongst a more limited pool of people. Few longitudinal studies have been conducted in high-income countries [1,10,11] and to our knowledge none have characterized recurrent IID in older age groups. The IID2 Study presented a unique opportunity to determine the extent to which repeat infections occur in elderly people. We conducted a secondary analysis of data from the recent IID2 Study in the UK to compare the characteristics of individuals reporting one and more than one episode of IID, and investigate whether the number of previous IID episodes reported influences the subsequent rate of IID in different age groups.

## Methods

The IID2 Study has been described in detail elsewhere [6,12]. Briefly, the study included a population cohort consisting of 6836 participants of all ages recruited from the registered population of 88 general practices in the UK. Individuals with known, chronic causes of diarrhoea were excluded, as were those with terminal illness, severe mental impairment and non-English speakers for whom a suitable interpreter could not be identified. Participants were followed up weekly for symptoms of diarrhoea and/or vomiting for a period of up to 52 weeks between October 2007 and August 2009. Symptomatic individuals were asked to complete a questionnaire with details of clinical characteristics of disease and healthcare usage and to submit a stool specimen for microbiological examination. A case of IID was defined as loose stools or clinically significant vomiting lasting less than 2 weeks, in the absence of a known non-infectious cause, preceded by a symptom-free period of 3 weeks. Vomiting was clinically significant if it occurred more than once in a 24 h period and if it incapacitated the patient or was accompanied by other symptoms such as cramps or fever [1]. The study was granted a favourable ethics opinion by the NHS North West Research Ethics Committee (07/MRE08/5), and was approved by 37 NHS Research Management and Governance organisations for the general practices involved.

Participants were followed up for a median of 39 weeks (inter-quartile range: 27 – 45 weeks). The rate of UK-acquired IID, standardized to the age and sex distribution of the UK population, was 274 cases per 1000 person-years (95% CI: 254 to 296).

For the present analysis, we investigated the frequency of recurrent IID, whether recurrent IID clustered in certain groups of individuals, and whether a previous episode of IID was associated with a higher incidence of recurrent episodes. We included in the analysis both UK-acquired and travel-related episodes meeting the above case definition for IID.

### Analysis

#### Characteristics of participants with no episodes, single episodes and multiple episodes of IID

We compared participants who did not report any episodes of IID throughout their follow-up period with participants who reported one and more than one episode. We compared these three groups in terms of age distribution, sex, socioeconomic classification, and area-level deprivation and urban rural classification based on their postcode of residence. Age was categorized into five strata: <1 year, one to four years, five to 14 years, 15 to 64 years, and 65+ years. Although these groupings are coarse, we did not find substantial differences in IID incidence beyond the age of five years in our study [2]. We measured socioeconomic classification using the National Statistics – Socioeconomic Classification (NS-SEC) [13]. We used the χ^2^ test and associated p-value to identify potentially important differences in these factors between groups.

#### Rates of IID

Since participants were followed up for varying lengths of time, we next calculated rates of IID, with associated 95% confidence intervals (95% CIs), by strata of the above factors, taking into account individuals’ length of follow-up. In addition, we calculated rates of IID according to the number of previous episodes participants had experienced.

#### Effect of previous episodes on rate of recurrent IID

We then investigated whether a prior episode of IID was associated with an increased rate of IID using Cox regression modelling. We used a counting process approach as recommended by Guo *et al*. [14]. In this approach, each participant contributes to the person-time of observation, and can contribute multiple IID episodes, for as long as they are at risk and remain in the study. Our case definition stipulated that disease had to last <14 days and should be preceded by a symptom-free period of at least three weeks. Thus, participants were considered at-risk from the fourth week of follow-up (provided they had been symptom-free since enrolment) until their first episode. They were considered not at-risk for the duration of their illness and for a further three weeks, at which point they returned to the at-risk pool. Weeks with missing responses were assumed to be non-informative (that is, the incidence of IID on these weeks was the same as on observed weeks), but individuals were censored for the duration of non-response and a further three weeks thereafter. Participants with symptoms of 14+ days duration were not included as IID cases, and were censored until three weeks after cessation of symptoms.

Participants could therefore contribute multiple spells of follow-up, each ending either in exit from the study (due to end of follow-up, end of the study, or loss to follow-up), an episode of IID, or censoring (due to non-response, or symptoms not meeting the case definition). For each spell of follow-up, we created a variable denoting how many previous IID episodes the participant had experienced, and included this as the independent variable of interest in our Cox model. We constructed a multivariable regression model using a forward stepwise approach. We adjusted in turn for age group, sex, NS-SEC, area-level deprivation and urban–rural classification. We looked for evidence of confounding by each of these factors by assessing their effect on estimates of the hazard ratio (HR) for the association between previous episodes and IID rates, and assessed their contribution to the model using the likelihood ratio (LR) test. Factors that resulted in a considerable change in the HR (>10%), or that resulted in a low LR test p-value (<0.05), were retained as covariates in the model. We additionally tested for evidence of a linear effect of the number of previous episodes on IID incidence. For this, we used the LR test to compare a model including indicators for the number of previous episodes with one including a single linear term, and opted for the model with the best fit. Finally, we investigated whether the effect of previous episodes on IID incidence was influenced by age group, by comparing models with and without an interaction term between these two variables using the LR test.

A complication of multiple-event data is the dependence of observations within individuals. This dependence is partly accounted for by the inclusion of number of previous episodes as a covariate in the model, as individuals with recurrent episodes must, by definition, have experienced at least one previous episode. However, because the LR test does not adequately deal with this dependence, we repeated the model selection process using robust standard errors and favouring models with lower values of the Akaike Information Criterion (AIC). Both model selection procedures resulted in the same final model.

### Sensitivity analysis

Incidence in the cohort decreased with time in study [2]. For this reason, we conducted a sensitivity analysis by sequentially excluding from our final model individuals with varying lengths of follow-up to determine whether this influenced the model coefficients.

Analysis was conducted using Stata 12.1 (Statacorp, Texas).

## Results

### Characteristics of participants with no episodes, single episodes and multiple episodes of IID

Among the 6836 participants, a total of 1320 IID cases occurred in 4658.6 person-years of follow-up. Nine hundred and one participants reported one episode of IID and 189 reported more than one. Infants and children under five years were more likely than older age groups to report at least one episode of IID (p < 0.001); 21% of infants experienced more than one IID episode (Table 1). Differences were seen for other factors, but no clear patterns emerged.

**Table 1:** Characteristics of cohort participants with 0, 1 and multiple episodes of IID during their follow-up time, UK 2008-9

	**Number of episodes**							
	**None**		**Single**		**Multiple**			
**Variable**	**No.**	**%**	**No**	**%**	**No**	**%**	**χ**^**2**^	**p**
Age group								
*<1 year*	24	57.1%	9	21.4%	9	21.4%	180.20	<0.001
*1 to 4 years*	182	62.5%	86	29.6%	23	7.9%		
*5 to 14 years*	517	82.9%	90	14.4%	17	2.7%		
*15 to 64 years*	3,357	84.5%	518	13.0%	99	2.5%		
*65+ years*	1,666	87.5%	198	10.4%	41	2.2%		
Sex								
*Male*	2,307	86.2%	300	11.2%	69	2.6%	15.92	<0.001
*Female*	3,439	82.7%	601	14.4%	120	2.9%		
NS-SEC^1^								
*Managerial and professional occupations*	2,938	82.6%	514	14.5%	105	3.0%	30.32	0.001
*Intermediate occupations*	256	83.1%	47	15.3%	5	1.6%		
*Small employers and own account workers*	574	82.7%	96	13.8%	24	3.5%		
*Lower supervisory and technical occupations*	620	89.5%	63	9.1%	10	1.4%		
*Semi-routine and routine occupations*	408	86.4%	53	11.2%	11	2.3%		
*Not classifiable*	950	85.4%	128	11.5%	34	3.1%		
Quintile of deprivation^2^								
*1 (most deprived)*	420	87.1%	49	10.2%	13	2.7%	12.48	0.131
*2*	644	86.2%	80	10.7%	23	3.1%		
*3*	1,515	83.3%	247	13.6%	56	3.1%		
*4*	1,798	83.9%	286	13.4%	58	2.7%		
*5 (least deprived)*	1,366	83.1%	239	14.5%	39	2.4%		
Urban/rural indicator^2^								
*Urban*	3,445	84.5%	514	12.6%	116	2.8%	15.06	0.005
*Town*	766	86.3%	96	10.8%	26	2.9%		
*Rural*	1,532	81.9%	291	15.6%	47	2.5%		

^1^ National-Statistics Socioeconomic Classification; ^2^ 3 missing values.

### Rates of IID

The overall rate of IID was 283.4 cases per 1000 person-years (95% CI: 268.5 - 299.1). The rate increased from 254.7 cases per 1000 person-years (95% CI: 240.0 - 270.3) among those with no previous IID episodes to 980.2 per 1000 person-years (95% CI: 719.0 - 1,336.3) among those with two previous episodes. Participants who experienced three or more previous episodes had even higher rates, although there were very few cases in these categories. IID incidence was also higher among infants and children under five years and females. Some differences were also observed between categories of NS-SEC, area-level deprivation and urban–rural classification (Table 2).

**Table 2:** Rates of IID in the community by age group, sex, socioeconomic and geographic factors, and number of previous IID episodes, IID2 Study, UK 2008-9

**Stratum**	**Cases**	**PY**	**Rate**	**(95% CI)**
All	1,320	4,658.6	283.4	(268.5 - 299.1)
Age group				
*<1 year*	33	26.9	1,228.3	(873.2 - 1,727.7)
*1 to 4 years*	138	190.8	723.4	(612.2 - 854.7)
*5 to 14 years*	129	424.1	304.2	(256.0 - 361.5)
*15 to 64 years*	728	2,647.8	274.9	(255.7 - 295.7)
*65+ years*	292	1,369.1	213.3	(190.2 - 239.2)
Sex				
*Male*	459	1,840.6	249.4	(227.6 - 273.3)
*Female*	861	2,818.0	305.5	(285.8 - 326.6)
Number of previous episodes				
*0*	1,086	4,300.0	254.7	(240.0 - 270.3)
*1*	186	349.6	532.1	(460.9 - 614.3)
*2*	40	40.8	980.2	(719.0 - 1,336.3)
*3*	7	4.1	1,710.2	(815.3 - 3,587.3)
*4*	1	0.1	7,305.0	(1,029.0 - 51,858.7)
Deprivation quintile				
*1 (most deprived)*	76	328.9	231.1	(184.5 - 289.3)
*2*	134	509.5	263.0	(222.1 - 311.6)
*3*	377	1,232.6	305.9	(276.5 - 338.3)
*4*	411	1,485.5	276.7	(251.2 - 304.8)
*5 (least deprived)*	322	1,100.4	292.6	(262.3 - 326.4)
NS-SEC^1^				
*Managerial and professional occupations*	749	2,418.3	309.7	(288.3 - 332.7)
*Intermediate occupations*	58	202.8	286.0	(221.1 - 370.0)
*Small employers and own account workers*	148	473.6	312.5	(266.0 - 367.1)
*Lower supervisory and technical occupations*	83	459.2	180.8	(145.8 - 224.2)
*Semi-routine and routine occupations*	78	320.6	243.3	(194.9 - 303.7)
*Not classifiable*	204	784.2	260.1	(226.8 - 298.4)
Urban–rural classification				
*Urban*	765	2,778.1	275.4	(256.5 - 295.6)
*Town*	158	580.2	272.3	(233.0 - 318.3)
*Rural*	397	1,298.6	305.7	(277.1 - 337.3)

^1^ National-Statistics Socioeconomic Classification.

### Effect of previous episodes on recurrent IID

The final model included age group, a linear term for the number of previous episodes, and their interaction, adjusted additionally for sex and NS-SEC. The interaction term was well supported by the LR test (p < 0.001), and this model gave the lowest AIC of all candidate models evaluated.

Among those with no previous episodes, infants had the highest rate of IID, five times higher than those aged 65+ years (HR = 5.0, 95% CI: 3.11 - 8.03) (Table 3). Among infants with at least one episode, each additional episode increased the rate of recurrent IID by 1.7 times on average (HR = 1.6, 95% CI: 1.25 – 2.32). By contrast, the elderly had the lowest rates of disease overall, but each additional episode resulted in a three-fold increase in the incidence of a subsequent episode (HR = 3.08, 95% CI: 2.54 - 3.74). The corresponding HRs were 2.49 among five to 14 year-olds, and 2.08 among 15 to 64 year-olds. Among one to five year-olds, there was very weak evidence for an effect of previous episodes on recurrent IID (HR = 1.49, 95% CI: 0.95 - 1.72).

**Table 3:** Factors associated with IID incidence in the community, IID2 Study, UK 2008–9

**Variable**	**RR**	**p**	**(95% CI)**^**1**^
Age group			
*<1 year-olds*	5.00	<0.001	(3.11 - 8.03)
*1 to 4 year-olds*	3.62	<0.001	(2.91 - 4.52)
*5 to 14 year-olds*	1.40	0.003	(1.12 - 1.76)
*15 to 64 year-olds*	1.27	0.002	(1.09 - 1.48)
*65+ year-olds*	*1.00*	*--*	--
Linear effect of previous episodes on:			
*<1 year-olds*	1.70	0.001	(1.25 - 2.32)
*1 to 4 year-olds*	1.28	0.109	(0.95 - 1.72)
*5 to 14 year-olds*	2.49	<0.001	(1.78 - 3.47)
*15 to 64 year-olds*	2.08	<0.001	(1.80 - 2.41)
*65+ year-olds*	3.08	<0.001	(2.54 - 3.74)
Sex			
*Male*	*1.00*	*--*	--
*Female*	1.22	0.001	(1.08 - 1.37)
NS-SEC			
*Managerial and professional occupations*	*1.00*	*--*	--
*Intermediate occupations*	0.96	0.772	(0.74 - 1.25)
*Small employers and own account workers*	0.98	0.843	(0.82 - 1.17)
*Lower supervisory and technical occupations*	0.63	<0.001	(0.51 - 0.80)
*Semi-routine and routine occupations*	0.82	0.089	(0.64 - 1.03)
*Not classifiable*	0.90	0.189	(0.76 - 1.05)

^1^95% CIs are based on robust standard errors.

Results from a multivariable Cox regression model.

### Sensitivity analysis

**Figure 1:**
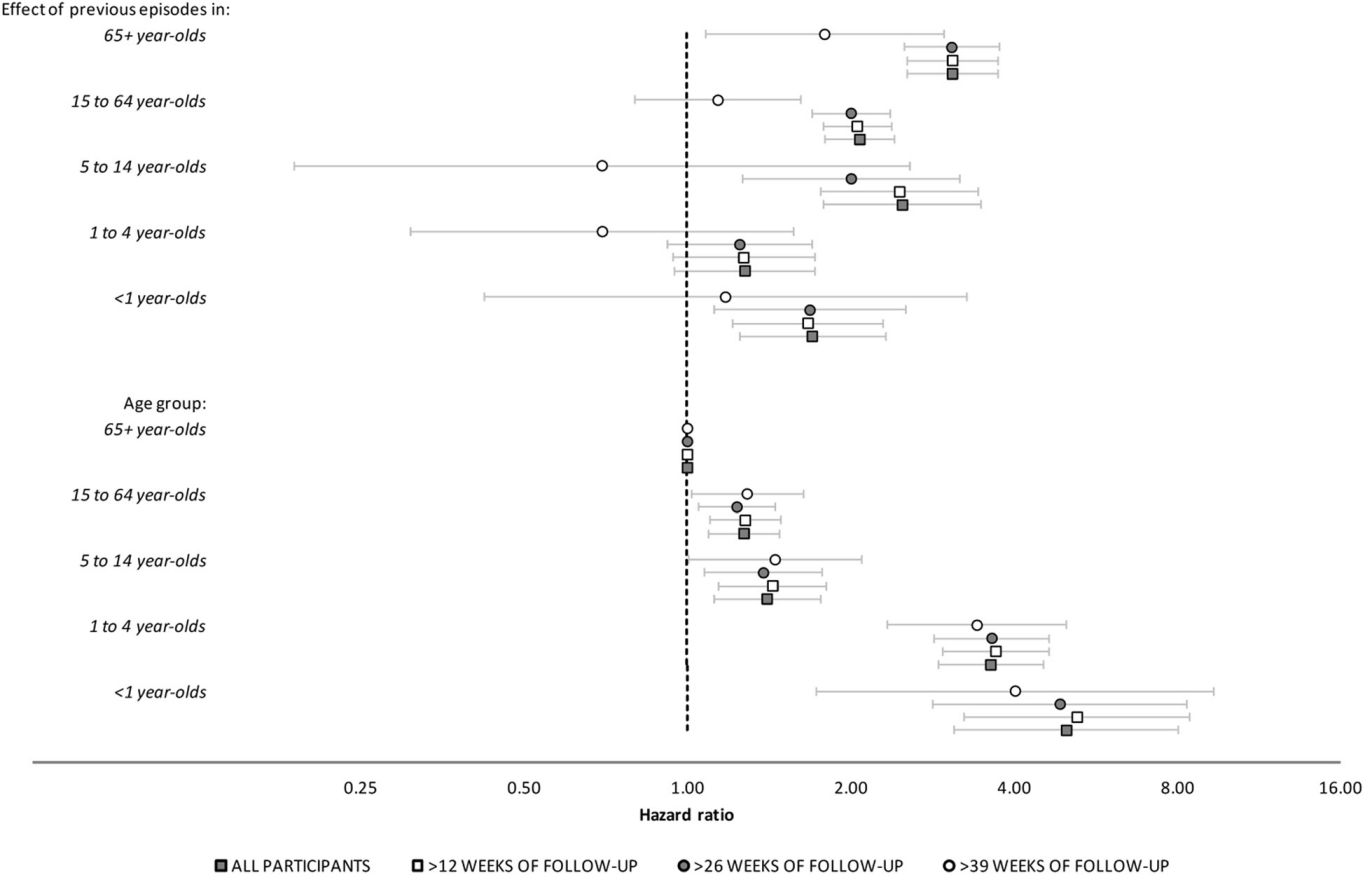
**Sensitivity analysis investigating the influence of varying lengths of follow-up on model-estimated hazard ratios and 95% CIs for the main effects of age and the interaction between age group and a linear effect of previous episodes.** Dark squares: all cohort participants; Light squares: participants with >12 weeks of follow-up; Dark circles: participants with >26 weeks of follow-up; Light circles: participants with >39 weeks of follow-up.

In the sensitivity analysis, excluding individuals with fewer than 27 weeks of follow-up had little effect on the results (Figure 1). When only individuals with >39 weeks of follow-up were included in the model, the effect of previous episodes on IID rate was markedly decreased in all age groups, although the main effect for age remained unchanged. This was likely the result of a loss of statistical power to investigate interaction effects, as there were no individuals with more than two episodes among those with >39 weeks of follow-up. The effect of previous episodes among the 65+ age group, however, was still apparent.

## Discussion and conclusion

Our results provide evidence that the rate of IID recurrence varies by age. Although infants experience more episodes of IID, among older age groups recurrent IID clusters much more strongly in certain individuals. This effect was particularly pronounced in the elderly, in whom every additional IID episode increased the rate of a subsequent episode by three times. We are not aware of previous evidence from observational studies for such strong clustering effects in adult age groups. In an analysis of surveillance data on laboratory-confirmed campylobacteriosis in Québec, Canada, Arsenault *et al.* found that, over a period of 11 years, a second episode of *Campylobacter* IID was more likely to be reported among those aged 15 years and above compared with children under 5 years [15].

While our data demonstrate an age-dependent effect of recurrent IID, we have no direct evidence for the biological mechanisms involved. However, it is possible that this effect results from changes in immune development and host susceptibility with age. For many enteric pathogens, rates of disease are highest in infants and decrease markedly with age. In particular, rotavirus is the third most common pathogen among paediatric IID cases [6], accounting for a sixth of cases in children under five years in the IID2 Study for whom an aetiological agent could be identified. Infection with rotavirus is known to decrease the risk of clinical disease following subsequent rotavirus infection [16,17]; by the age of five, most infections are asymptomatic. This would partly account for the decreased effect of previous episodes on recurrent disease in young children compared with other age groups. Infection with norovirus and sapovirus, the two commonest pathogens in this age group, results in short-term protection from re-infection with related strains, and could also explain the lower rate of recurrent disease in young children compared with older age groups over the relatively short follow-up period of our study.

Among older individuals, the increased rate of recurrent IID is likely due to a greater proportion of individuals with impaired immune function, or chronic or transient gastrointestinal conditions that increase susceptibility to infection with enteric pathogens. Alternatively, infection at older age groups could result in long-lasting changes to the gut flora that facilitate subsequent infection. In our study, we excluded individuals with known chronic gastrointestinal conditions, but it is possible that a fraction of individuals had undiagnosed bowel abnormalities. We did not have information on other conditions that affect immune function, such as diabetes and HIV, or on the use of medications that could influence susceptibility to infection, such as use of steroids, antibiotics, or proton pump inhibitors.

An alternative explanation for the higher rates of recurrent IID in older age groups is that enteric infections themselves increase the risk of bowel abnormalities; irritable bowel syndrome is commonly reported following infection with *Campylobacter* and other bacterial pathogens [18,19]. This could suggest that infection with specific pathogens might lead to subsequent diarrhoea episodes in which no pathogen can be found. Our study was insufficiently powered to investigate the effect of specific pathogens on subsequent IID episodes; 60% of specimens in the IID2 Study were negative for any of the pathogens investigated [6], and the number of recurrent episodes was relatively small. However, we defined a recurrent episode as one that was preceded by a three-week symptom-free period, and our case definition excluded episodes of diarrhoea lasting more than 14 days. It is therefore unlikely that these recurrent episodes constituted persistent or pre-existing bowel abnormalities.

Ascertainment of diarrhoea cases in the IID2 Study relied on weekly, active follow-up of individuals and included negative reporting of symptoms. Nevertheless, it is possible that completeness of reporting, or the effects of reporting fatigue, differed between age groups. This could partly explain our results if reporting of symptoms was more complete in older age groups, but decreased over the follow-up period among younger individuals. In our data, this would manifest itself as a change in the relative rate of disease between different age groups over time, effectively a violation of the proportional hazards assumption required by the Cox model. We found no evidence of a violation of this assumption, and we think this explanation is unlikely.

A further limitation of our study is that we only had information on IID symptoms during the follow-up period. Our data on previous IID episodes is therefore limited to the period of observation. This is likely to underestimate individuals’ IID experience and would tend to limit our ability to observe an effect on recurrent IID, although it is unlikely to have resulted in spurious associations.

Our study indicates that certain subgroups in the population have a high propensity for recurrent IID, particularly among older age groups. The mechanisms for this are unclear, but prevention and control of IID is likely to require a better understanding of how underlying conditions affect both the risk and the outcomes of IID, especially among the elderly. More detailed studies among vulnerable populations, including those with underlying conditions or impaired immunity, should help to better establish the risks and pathogens associated with certain subgroups and inform adequate control policies. Clinicians should be aware of the increased risk of recurrent IID among the elderly and consider whether closer monitoring is required, particularly in the context of underlying conditions. Diarrhoea is a common condition in the elderly that can have profound consequences either owing to the effects of the causative organism itself or because of the associated dehydration [20,21]. *C. difficile* is considered to be the most likely cause of persistent or relapsing diarrhoea in the elderly, but our analysis has shown that recurrent IID was common in an elderly population in which *C. difficile* was rare. With the advent of molecular methods that can be used to screen for multiple pathogens at the same time it is possible that the diversity of organisms causing recurrent gastroenteritis in the elderly will be better understood. This, in turn, might mean that unnecessary antibiotic treatment or invasive procedures can be avoided.

## Competing interests

The authors declare that they have no competing interests.

## Authors’ contributions

CCT conceived the study and performed the analysis. LV contributed to the study design. CCT, LV, LCR and SJO’B contributed to the drafting of the manuscript, and read and approved the final version.

## Pre-publication history

The pre-publication history for this paper can be accessed here:

http://www.biomedcentral.com/1471-2458/13/739/prepub
